# Structure of fumarate hydratase from *Rickettsia prowazekii*, the agent of typhus and suspected relative of the mitochondria

**DOI:** 10.1107/S174430911102690X

**Published:** 2011-08-16

**Authors:** Isabelle Phan, Sandhya Subramanian, Christian Olsen, Thomas E. Edwards, Wenjin Guo, Yang Zhang, Wesley C. Van Voorhis, Lance J. Stewart, Peter J. Myler

**Affiliations:** a Seattle Structural Genomics Center for Infectious Diseases (SSGCID), USA; b Seattle Biomedical Research Institute, USA; c Emerald BioStructures, USA; d Departments of Microbiology, Genome Sciences and Immunology, University of Washington, USA

**Keywords:** tricarboxylic acid cycle, mitochondria, *Rickettsia*, typhus, fumarate hydratases, lyases

## Abstract

Rickettsiae are obligate intracellular parasites of eukaryotic cells that are the causative agents responsible for spotted fever and typhus. Their small genome (about 800 protein-coding genes) is highly conserved across species and has been postulated as the ancestor of the mitochondria. No genes that are required for glycolysis are found in the *Rickettsia prowazekii* or mitochondrial genomes, but a complete set of genes encoding components of the tricarboxylic acid cycle and the respiratory-chain complex is found in both. A 2.4 Å resolution crystal structure of *R. prowazekii* fumarate hydratase, an enzyme catalyzing the third step of the tricarboxylic acid cycle pathway that ultimately converts phospho­enolpyruvate into succinyl-CoA, has been solved. A structure alignment with human mitochondrial fumarate hydratase highlights the close similarity between *R. prowazekii* and mitochondrial enzymes.

## Abstract 2

Fumarate hydratase is an enzyme of the tricarboxylic acid cycle, one of the metabolic pathways characteristic of the mitochondria. The structure of *R. prowazekii* class II fumarate hydratase is reported at 2.4 Å resolution and is compared with the available structure of the human homolog.

## 1. Introduction

Typhus epidemics have been recurrent in human history; the pattern of infection was such that the bacterium *Rickettsia prowazekii*, the agent of typhus, could arguably determine the outcome of war, with outbreaks after World War I resulting in around three million deaths (Raoult *et al.*, 2004 ▶). Although hecatombs of this scale remain exceptional, typhus continues to ravage populations in areas of conflict, with mortality rates among infected patients of as high as 20% without antibiotics (Center for Biosecurity of UPMC; http://upmc-biosecurity.org). Despite its biological characteristics (environmental stability, small size, aerosol transmission, persistence in infected hosts, low infectious dose, high morbidity and substantial mortality), *R. prowazekii* may not be a primary bioweapon candidate because of its dependency on its eukaryotic host for propagation (Azad, 2007 ▶), although this view remains disputed (Walker, 2009 ▶). Nonetheless, the Center for Disease Control and Prevention (CDC) ranks *R. prowazekii* as a Category B biological agent and the Department of Health and Human Services (DHHS) classifies it as a top priority for the development of medical countermeasures, thus further encouraging efforts to understand the mechanism of action of this pathogen.

The complete genome of *R. prowazekii* contains only 834 protein-coding genes, a very small number compared with the 5000 genes found in the model bacterium *Escherichia coli*, highlighting simil­arities between *R. prowazekii* and mitochondrial genes as well as the absence of the genes required for anaerobic glycolysis. It has been suggested that ATP production in *Rickettsia* is the same as that in mitochondria (Andersson *et al.*, 1998 ▶). Despite the difference in size between the *Rickettsia* genome (over 1 000 000 bp) and that of human mitochondrial DNA (16 000 bp), the results of phylogenetic studies are consistent with an α-proteobacterial ancestry of the mitochondrial genome (Gray *et al.*, 2001 ▶). However, comparisons at the protein level reveal a far more complex picture, since 90% of the mitochondrial proteins are encoded in the nucleus (Gray *et al.*, 2004 ▶). One such example is fumarate hydratase, a mitochondrial enzyme from the citric acid cycle, which is encoded on nuclear chromosome 1 in humans (Craig *et al.*, 1976 ▶).

**Figure 1:**
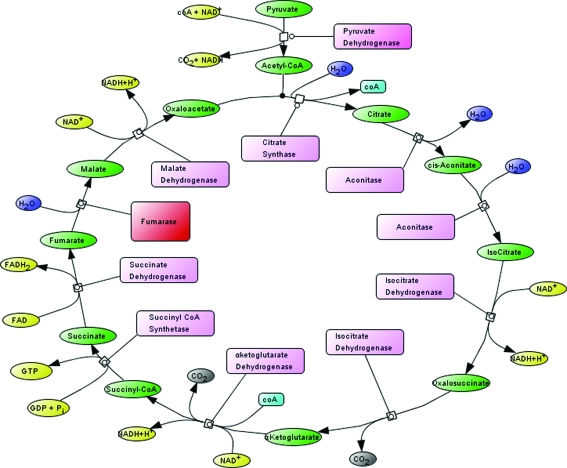
Chemical reaction pathway of the tricarboxylic acid cycle (TCA; also known as the Krebs cycle and the citric acid cycle); catalytic enzymes are indicated in pink boxes, with fumarase, the subject of this study, highlighted in red. This figure was prepared with *CellDesigner* (Funahashi *et al.*, 2003 ▶).

The tricarboxylic acid cycle (TCA; also known as the Krebs cycle and the citric acid cycle) is a pathway that Tyler described in 1992 as ‘so crucial to the metabolism of living cells that any significant defect is incompatible with life’ (Tyler, 1992 ▶). The cycle is constituted by a series of biochemical reactions that lead to the progressive oxidative decarboxylation of acetyl-CoA (see Fig. 1 ▶). The step that converts fumarate to l-malate has recently been the target of studies of tumorigenesis in humans (King *et al.*, 2006 ▶) and pathogenicity in bacteria (van Ooij, 2010 ▶).

Two classes of enzymes, class I and class II fumarate hydratase (fumarase; FumC), reversibly convert fumarate to l-malate and have no detectable sequence similarity (Woods *et al.*, 1988 ▶). Class I fumarases (FumA and FumB enzymes) are homodimeric, thermolabile, iron–sulfur-containing enzymes of approximately 120 kDa. Class II fumarases (FumC enzymes) are homotetrameric, thermostable, iron-independent enzymes with a molecular mass of 200 kDa. The amino-acid sequences of mitochondrial class II FumCs are highly conserved in eukaryotes and are most closely related to the α-­proteobacterial homologues (Schnarrenberger & Martin, 2002 ▶).

Defects in human FumC are the cause of fumarase deficiency, a disease characterized by progressive encephalopathy, developmental delay, hypotonia, cerebral atrophy and lactic and pyruvic acidemia (Coughlin *et al.*, 1998 ▶). Heterozygous germline mutations of FumC were found in patients with multiple cutaneous and uterine leiomyomas (MCUL). A further set of mutations is the cause of hereditary leiomyomatosis and renal cell cancer (HLRCC). Research to elucidate the mechanisms that lead to enhanced glycolysis in tumours has shown that FumC and succinate dehydrogenase (SDH) are tumour suppressors, demonstrating for the first time how mitochondrial enzymes and their dysfunction are associated with tumori­genesis (King *et al.*, 2006 ▶). A dedicated online database of FumC gene mutations lists all reported FumC sequence variants (Bayley *et al.*, 2008 ▶).

Besides its involvement in human tumorigenesis, the TCA cycle has been targeted for its role in pathogenicity. In particular, FumC was found to be one of nine *in vivo*-induced virulence factors in *Listeria* (Wilson *et al.*, 2001 ▶) and to bind PdhS, an essential cytoplasmic histidine kinase involved in differentiation, in *Brucella* (Mignolet *et al.*, 2010 ▶). A recent paper further shows that the TCA cycle signals the switch between a pathogenic state and a mutualistic state when the *Photorhabdus* bacterium changes hosts (Lango & Clarke, 2010 ▶).

To this day, the SSGCID project is the sole depositor of *Rickettsia* structures in the Protein Data Bank. Here, we present the high-resolution structure of *R. prowazekii* FumC and compare it with that of its human mitochondrial homolog.

## 2. Methods

### 2.1. Protein expression and purification

FumC from *R. prowazekii* strain Madrid E (NCBI NP_221027; *fumC* gene; EC 4.2.1.2; UniProt Q9ZCQ4) spanning the full-length protein from residues 1–461 (‘ORF’) was cloned into the ligation-independent cloning (LIC; Aslanidis & de Jong, 1990 ▶) expression vector pAVA0421 encoding an N-terminal hexahistidine affinity tag followed by the human rhinovirus 3C protease cleavage sequence (MAHHHHHHMGTLEAQTQGPGS-ORF).

The construct encoding the gene for FumC was transformed into chemically competent *E. coli* BL21 (DE3) Rosetta cells. An overnight culture was grown in LB broth at 310 K and was used to inoculate 2 l ZYP-5052 auto-induction medium, which was prepared as described by Studier (2005 ▶). FumC was expressed in a LEX bioreactor in the presence of antibiotics. After 24 h at 298 K, the temperature was reduced to 288 K for a further 60 h. The sample was centrifuged at 4000*g* for 20 min at 277 K and the cell paste was flash-frozen in liquid nitrogen and stored at 193 K.

During the purification process, the frozen cell pellet was thawed and completely resuspended in lysis buffer (20 m*M* HEPES pH 7.4, 300 m*M* NaCl, 5% glycerol, 30 m*M* imidazole, 0.5% CHAPS, 10 m*M* MgCl_2_, 3 m*M* β-mercaptoethanol, 1.3 mg ml^−1^ protease-inhibitor cocktail and 0.05 mg ml^−1^ lysozyme). The resuspended cell pellet was then disrupted on ice for 15 min with a Branson Digital 450D Sonifier (70% amplitude, with alternating cycles of 5 s pulse-on and 10 s pulse-off). The cell debris was incubated with 20 µl Benzonase nuclease at room temperature for 40 min. The lysate was clarified by centrifugation with a Sorvall RC5 at 10 000 rev min^−1^ for 60 min at 277 K in a F14S Rotor (Thermo Fisher). The clarified solution was syringe-filtered through a 0.45 µm cellulose acetate filter (Corning Life Sciences, Lowell, Massachusetts, USA). The lysate was purified by IMAC using a HisTrap FF 5 ml column (GE Biosciences, Piscataway, New Jersey, USA) equilibrated with binding buffer (25 m*M* HEPES pH 7.0, 300 m*M* NaCl, 5% glycerol, 30 m*M* imidazole, 1 m*M* TCEP) and eluted with 500 m*M* imidazole in the same buffer. The eluted FumC was concentrated and further resolved by size-exclusion chromatography (SEC) using a Superdex 75 26/60 column (GE Biosciences) equilibrated in SEC buffer (20 m*M* HEPES pH 7.0, 300 m*M* NaCl, 5% glycerol and 1 m*M* TCEP) attached to an ÄKTA FPLC system (GE Biosciences). Peak fractions were collected and pooled based on purity-profile assessment by SDS–PAGE. Concentrated pure protein was flash-frozen in liquid nitrogen and stored at 193 K. The final concentration (39.5 mg ml^−1^) was determined by UV spectrophotometry at 280 nm using a molar extinction coefficient of 33 015 *M* ^−1^ cm^−1^ and the final purity (>97%) was assayed by SDS–PAGE.

### 2.2. Crystallization

Crystallization trials were set up according to a rational crystallization approach (Newman *et al.*, 2005 ▶) using the JCSG+ and PACT sparse-matrix screens from Emerald BioSystems and Molecular Dimensions. Protein (39.5 mg ml^−1^, 0.4 µl) in SEC buffer (20 m*M* HEPES pH 7.0, 300 m*M* NaCl, 5% glycerol and 1 m*M* TCEP) was mixed with an equal volume of precipitant and equilibrated against an 80 µl reservoir in sitting-drop vapor-diffusion format in 96-­well Compact Jr plates from Emerald BioSystems at 289 K. Within six weeks, crystals grew in the presence of 2.4 *M* sodium malonate (JCSG+ condition F9). A gradient optimization screen was designed based on this condition and crystals grew from this screen after about six weeks in 1.4 *M* sodium malonate pH 6.0.

### 2.3. Data collection and structure determination

A crystal was harvested, cryoprotected with a solution consisting of the precipitant supplemented with 20% glycerol and vitrified in liquid nitrogen. A 2.4 Å resolution data set was collected at the Advanced Light Source (Andersson *et al.*, 1998) on beamline 5.0.2 (Table 1 ▶). The data were reduced with *XDS*/*XSCALE* (Kabsch, 2010 ▶). The structure was determined by molecular replacement using human FumC (PDB entry 3e04; Structural Genomics Consortium, unpublished work) as a search model in *Phaser* (McCoy *et al.*, 2007 ▶) from the *CCP*4 suite (Winn *et al.*, 2011 ▶). The refinement statistics are shown in Table 2 ▶. The asymmetric unit contained two protomers of the biologically relevant tetramer, with the other two protomers being generated by crystallographic symmetry. The final model was obtained after numerous iterative rounds of refinement in *REFMAC* (Murshudov *et al.*, 2011 ▶) and manual rebuilding in *Coot* (Emsley & Cowtan, 2004 ▶). The final model consisted of residues Asn3–Glu457 with no internal gaps for protomer *A*, residues Asn3–Pro316 and Met321–Leu406 for protomer *B*, 198 water molecules, two malonate molecules (one bound to each protomer) and a sodium ion assigned based on the crystallization conditions (sodium malonate), *B* factors and coordination distances of ∼2.5 Å (Zheng *et al.*, 2008 ▶). The structure was assessed and corrected for geometry and fitness using *MolProbity* (Chen *et al.*, 2010 ▶).

## 3. Discussion

**Figure 2:**
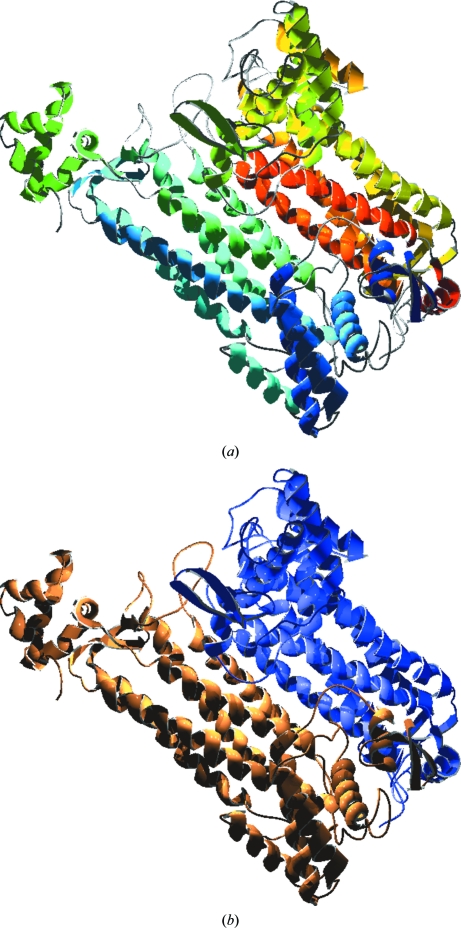
Ribbon diagram of the homodimeric unit structure of *R. prowazekii* FumC showing (*a*) the overall fold gradient-coloured from red (N-terminus) to blue (C-­terminus) and (*b*) the backbone trace of chain *A* (blue) and chain *B* (brown) of the dimeric unit. This figure and all other structure figures in this paper (except for Fig. 5 ▶) were prepared using the *POV-Ray* renderer (http://povray.org) and *DeepView* (Guex & Peitsch, 1997 ▶).

**Figure 3:**
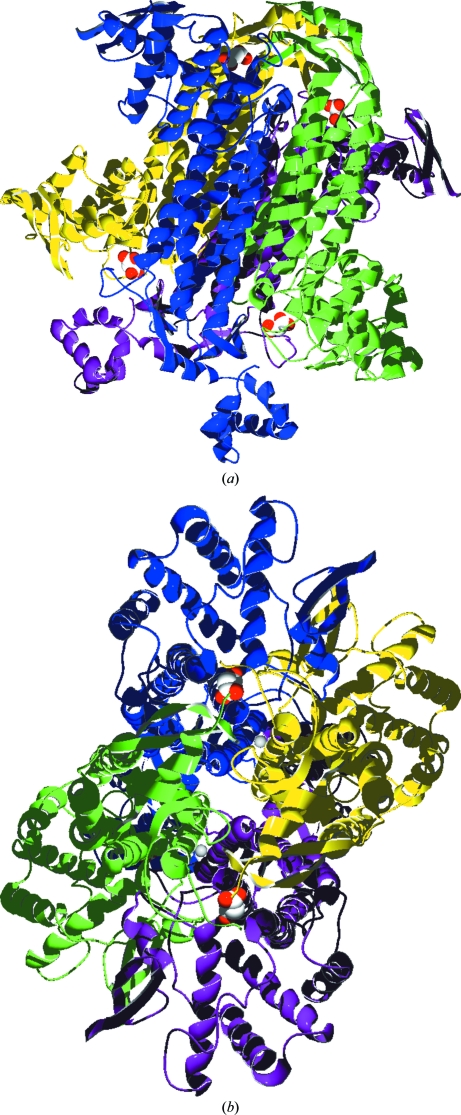
Two views of the tetrameric assembly predicted by *PISA* from the 3gtd coordinates (http://www.ebi.ac.uk/msd-srv/prot_int/pistart.html) showing the schematic backbone trace of the four subunits modelled for dimer 1 chain *A* (blue) and *B* (yellow) and dimer 2 chain *A* (magenta) and *B* (green). (*a*) The side view of each chain bound to the ligand malonate shown in CPK. (*b*) The two sodium ions at the interface of each dimer can be seen near the central axis of symmetry.

The *R. prowazekii* FumC structure was determined in complex with the product analog malonate. Like the first reported FumC structure from *E. coli* (Weaver *et al.*, 1995 ▶), *R. prowazekii* FumC crystallized as a homodimer containing two subunits of the normally tetrameric enzyme (see Fig. 2 ▶), in which each chain forms an elongated central four-helix bundle capped by two compact domains at the N- and C-­termini. Fig. 3 ▶ shows the tetrameric assembly predicted by the *PISA* quaternary-structure tool (Krissinel & Henrick, 2007 ▶), including the malonate ligand in the active site.

**Figure 4:**
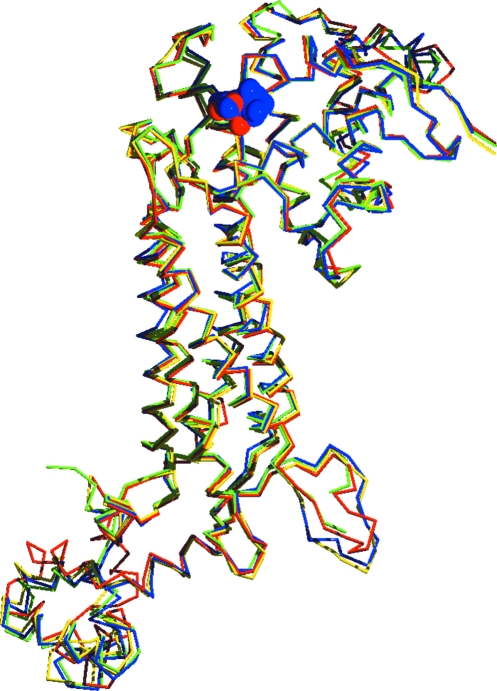
Superposition of the backbone C^α^ traces of the FumC monomers from *Rickettsia* (PDB entry 3gtd; red), human (PDB entry 3e04; green; Structural Genomics Consortium, unpublished work), *E. coli* (PDB entry 1kq7; blue; Estévez *et al.*, 2002 ▶) and *S. cerevisiae* (PDB entry 1yfm; yellow; Weaver *et al.*, 1998 ▶) showing the conserved overall fold and the deviations at the C-terminus at the bottom left region of the structure. The ligands for the *Rickettsia* and *E. coli* structures, malonate (red) and citric acid (blue), respectively, are represented in CPK colors.

**Figure 5:**
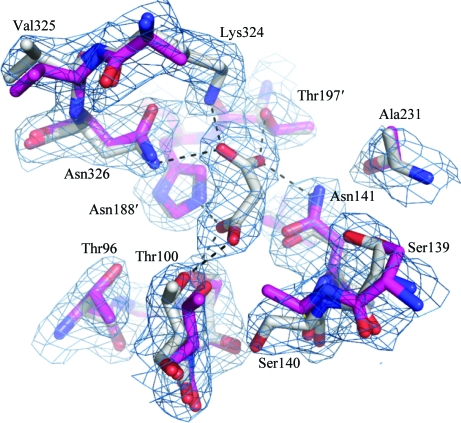
Schematic representation of the ligand environment in the *Rickettsia* FumC monomer complexed with malonate superimposed on the corresponding residues in the human structure (PDB entry 3e04). The backbone (gray for *Rickettsia*, magenta for human) and side chains of residues located within 6 Å of the ligand are shown. Hydrogen bonds of less than 3 Å are shown as dashed lines. Residues are numbered according to the *Rickettsia* FumC numbering system and residues from the second protomer that comprise the active site are identified with the ′ notation (Thr187′ and His188′). The 2|*F*_o_| − |*F*_c_| electron-density map is shown in light blue mesh contoured at 1.0σ. Note that the human structure is apo and thus Lys371 (equivalent to Lys324 in *Rickettsia*) appears disordered. This figure was generated using *PyMOL* (DeLano, 2002 ▶).

Structure alignment of the *R. prowazekii* FumC monomer with the human enzyme using *MultiProt* (Shatsky *et al.*, 2004 ▶) showed an r.m.s.d. of 0.99 Å over 92% of the sequence. The average C^α^ r.m.s.d. of a global alignment of FumC structures from *Rickettsia* (bound to the product analog malonate in the active site), human (unbound; PDB entry 3e04; Structural Genomics Consortium, unpublished work), *E. coli* (bound to the competitive inhibitor citrate in the active site and to *S*-malate in the B site; Estévez *et al.*, 2002 ▶) and *Saccharo­myces cerevisiae* (unbound; Weaver *et al.*, 1998 ▶) is 0.90 Å over 410 residues (see Table 3 ▶ for pairwise r.m.s.d.s). The largest deviation is found in the C-­terminal region; otherwise the backbone structure is remarkably conserved, including the active site (see Fig. 4 ▶). The residues located within 6 Å of the ligand in the *Rickettsia* structure, Thr96, Ser98, Ser139, Ser140, Asn141, Ala231 and Leu358, are 100% conserved in the three other species and adopt almost identical conformations, even in the unbound structures: the r.m.s.d. for all atoms over those eight residues is 0.83 Å from the human structure, 1.09 Å from that from *E. coli* and 1.15 Å from that from *S. cerevisiae*. The only visible difference between the human and *Rickettsia* pockets is the tilting of the Ser140 hydroxyl group away from the active site in the human structure (see Fig. 5 ▶).

FumC displays some essential features of a good drug target: it is clearly involved in a crucial biological pathway, is functionally well characterized and possesses a druggable binding site. However, the structural evidence obtained in the present study strongly indicates that this enzyme is an unsuitable target for therapeutic intervention against *Rickettsia* owing to the very high degree of conservation between the human and *R. prowazekii* structures in terms of both the global fold and the binding site.

## Supplementary Material

PDB reference: fumarate hydratase, 3gtd

## Figures and Tables

**Table 1:** Data-collection statistics Values in parentheses are for the highest of 20 resolution shells.

Space group	*P*3_1_21
Unit-cell parameters (Å)	*a* = *b* = 144.9, *c* = 106.21
Wavelength (Å)	1.0
Resolution range (Å)	19.74–2.4 (2.46–2.40)
No. of unique reflections	46831
Completeness (%)	99.7 (97.5)
*R*_merge_†	0.12 (0.73)
Mean *I*/σ(*I*)	9.5 (2.7)

†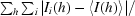 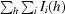.

**Table 2:** Refinement and model statistics Values in parentheses are for the highest of 20 resolution shells.

Resolution range (Å)	19.74–2.4 (2.46–2.40)
*R*_cryst_†	0.194
*R*_free_†	0.226
R.m.s.d. bonds (Å)	0.0080
R.m.s.d. angles (°)	1.043
Protein atoms	6571
Nonprotein atoms	213
Mean *B* factor (Å^2^)	20.275
Residues in favored region (%)	98
Residues in allowed region (%)	100
*MolProbity*‡ score [percentile]	1.4 [99th]

† *R*_cryst_ = 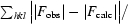 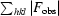. The free *R* factor was calculated using 5% of the reflections omitted from the refinement (Winn *et al.*, 2011 ▶).

‡ Chen *et al.* (2010 ▶).

**Table 3:** Best pairwise backbone C^α^ r.m.s.d. between the FumC structure from *Rickettsia* and those from human, *E. coli* and *S. cerevisiae* (yeast) calculated using *MultiProt* (Shatsky *et al.*, 2004 ▶)

Structure pairs	R.m.s.d. (Å)	Alignment size (residues)
3gtd (*Rickettsia*)/1kq7 (*E. coli*)	0.98	416
3gtd (*Rickettsia*)/1yfm (yeast)	0.98	418
3gtd (*Rickettsia*)/3e04 (human)	0.99	423
